# P2Y_2_ Receptor Induces *L. amazonensis* Infection Control in a Mechanism Dependent on Caspase-1 Activation and IL-1*β* Secretion

**DOI:** 10.1155/2020/2545682

**Published:** 2020-10-01

**Authors:** Maria Luiza Thorstenberg, Monique Daiane Andrade Martins, Vanessa Figliuolo, Claudia Lucia Martins Silva, Luiz Eduardo Baggio Savio, Robson Coutinho-Silva

**Affiliations:** ^1^Laboratório de Imunofisiologia, Instituto de Biofísica Carlos Chagas Filho, Universidade Federal do Rio de Janeiro, Rio de Janeiro, Brazil; ^2^Laboratório de Farmacologia Bioquímica e Molecular, Instituto de Ciências Biomédicas, Universidade Federal do Rio de Janeiro, Brazil

## Abstract

Leishmaniasis is a neglected tropical disease caused by an intracellular parasite of the genus *Leishmania*. Damage-associated molecular patterns (DAMPs) such as UTP and ATP are released from infected cells and, once in the extracellular medium, activate P2 purinergic receptors. P2Y_2_ and P2X7 receptors cooperate to control *Leishmania amazonensis* infection. NLRP3 inflammasome activation and IL-1*β* release resulting from P2X7 activation are important for outcomes of *L. amazonensis* infection. The cytokine IL-1*β* is required for the control of intracellular parasites. In the present study, we investigated the involvement of the P2Y_2_ receptor in the activation of NLRP3 inflammasome elements (caspase-1 and 11) and IL-1*β* secretion during *L. amazonensis* infection in peritoneal macrophages as well as in a murine model of cutaneous leishmaniasis. We found that 2-thio-UTP (a selective P2Y_2_ agonist) reduced parasite load in *L. amazonensis*-infected murine macrophages and in the footpads and lymph nodes of infected mice. The antiparasitic effects triggered by P2Y_2_ activation were not observed when cells were pretreated with a caspase-1 inhibitor (Z-YVAD-FMK) or in macrophages from caspase-1/11 knockout mice (CASP-1,11^−/−^). We also found that UTP treatment induced IL-1*β* secretion in wild-type (WT) infected macrophages but not in cells from CASP-1,11^−/−^ mice, suggesting that caspase-1 activation by UTP triggers IL-1*β* secretion in *L. amazonensis*-infected macrophages. Infected cells pretreated with IL-1R antagonist did not show reduced parasitic load after UTP and ATP treatment. Our *in vivo* experiments also showed that intralesional UTP treatment reduced both parasite load (in the footpads and popliteal lymph nodes) and lesion size in wild-type (WT) and CASP-11^−/−^ but not in CASP-1,11^−/−^ mice. Taken together, our findings suggest that P2Y_2_R activation induces CASP-1 activation and IL-1*β* secretion during *L. amazonensis* infection. IL-1*β*/IL-1R signaling is crucial for P2Y_2_R-mediated protective immune response in an experimental model of cutaneous leishmaniasis.

## 1. Introduction

Leishmaniasis is a vector-borne disease caused by flagellated protozoans of the genus *Leishmania*. This disease represents a spectrum of neglected tropical diseases that are endemic in 98 countries worldwide [1]. The clinical manifestations range from cutaneous or mucocutaneous lesions to lethal visceral pathology. Cutaneous leishmaniasis, whose symptoms range from local ulcers to mucosal tissue destruction, can be caused by *L. amazonensis*, *L. major*, *L. braziliensis*, and *L. guaynensis* [2].

In humans, *Leishmania* promastigotes are injected into the dermis (i.e., through the bite of an infected sandfly) and establish infection in phagocytic cells [3]. The recognition of pathogen-associated molecular patterns (PAMPs) by phagocytes leads to the release of damage-associated molecular patterns (DAMPs) such as the extracellular nucleotides ATP (eATP) and UTP (eUTP) that are involved in the killing of intracellular pathogens through the activation of P2 receptors ([4, 5]. P2 purinergic/pyrimidinergic receptors can be subdivided into metabotropic G-protein-coupled P2Y (P2Y_1,2,4,6,11–14_) and ionotropic P2X receptors (P2X1–7) [6]. The following agonists activate P2 receptors: P2X and P2Y_11_–ATP; P2Y_2,4_–ATP and –UTP; P2Y_1_, P2Y_12_, and P2Y_13_–ADP; P2Y_6_–UDP; and P2Y_14_–UDP–glucose [7]. P2Y receptors (P2YR) can be constitutively expressed or regulated under pathological conditions [8]. Metabotropic G-coupled-proteins such as calcium-sensing receptor and P2YR were reported to be implicated in NLRP3 inflammasome activation in inflammatory models [9–13].

Evidence supports the involvement of the noncanonical NLRP3 inflammasome assembly in the elimination of *L. amazonensis* infection by P2X7R triggering, acting as an important platform to improve host leishmanicidal mechanisms. Nevertheless, the mechanisms involved in the activation of this inflammasome in leishmaniasis remain elusive [14, 15]. ATP/P2X7R signaling during *L. amazonensis* infection has been partially elucidated during the last decade; it is assumed to be the most potent canonical activator of the NLRP3 inflammasome [16] and more recently in noncanonical activation of NLRP3 inflammasome assembly [14, 17, 18]. We previously demonstrated that the antiparasite immune response attributed to the P2YR agonist UTP involves paracrine activation of the P2X7 receptor (P2X7R) and PANX-1 channels in macrophages from mice infected with *L. amazonensis* [19]; UTP induces production and release of reactive oxygen species (ROS), nitrite oxide (NO) [20, 21], and leukotriene B_4_ (LTB_4_) [19] that is assumed to be crucial for parasite death. In addition to these inflammatory mediators, IL-1*β* mediates the control of intracellular parasite infections [4] [22]. In the present study, we investigated caspase-1/IL-1*β* axis activation in the protective immune response induced by P2Y_2_ receptor activation in an experimental model of cutaneous leishmaniasis.

## 2. Materials and Methods

### 2.1. Chemicals

UTP (uridine triphosphate), ATP (adenosine triphosphate), Dulbecco's modified Eagle's medium (DMEM), and 199 medium were purchased from Sigma-Aldrich (St. Louis, MO, USA); 2-thio-UTP and FMK-Z-YVAD were from Tocris (Bristol, UK).

### 2.2. Mice

The experiments, maintenance, and care of mice were carried out according to the guidelines of the Brazilian College of Animal Experimentation (COBEA). We used wild-type BALB/c and C57BL/6 (Jackson Laboratory, USA), as well as CASP-1,11 and CASP-11 knockout mice (CASP-1,11^−/−^ and CASP-11^−/−^ against the C57BL/6 background) (Genentech Laboratory, South San Francisco, CA, USA). CASP-1,11^−/−^ and CASP-11^−/−^ mice were maintained at the Laboratory of Transgenic Animals of the Federal University of Rio de Janeiro (UFRJ, RJ, Brazil). The mice were housed in a temperature-controlled room (22°C) with a light/dark cycle (12 h). Food and water were provided *ad libitum*. The animal experimentation protocols used in this study were approved by the Ethics Committee on the Use of Animals (CEUA) from the IBCCF, UFRJ, document no. 077/15.

### 2.3. Parasite Culture

We used *L. amazonensis* (MHOM/BR/Josefa) strain in both *in vitro* and *in vivo* experiments. Amastigotes isolated from mouse lesions (from BALB/c mice) were allowed to transform into axenic promastigote forms by growth at 24°C, for 7 days, in 199 medium supplemented with 10% heat-inactivated fetal bovine serum (FBS; Gibco BRL, 2% male human urine, 1% L-glutamine and 0.25% hemin). Promastigotes in the late stationary phase of growth until the tenth passage were used to preserve parasite virulence.

### 2.4. Cell Culture and Infection

Resident macrophages were harvested from the peritoneal cavity by washes with cold phosphate buffer saline (PBS). Cells were directly seeded on culture plates (in DMEM-supplemented medium, at 37°C, with 5% CO_2_) for 1 h and washed gently with PBS (twice) to remove nonadherent cells. The cells were cultured for 24 h in DMEM supplemented (10% FBS and 100 units penicillin/streptomycin) at 37°C (and 5% CO_2_) and infected for 4 h or 1 h with *L. amazonensis* promastigotes (MOI ratio 10 : 1, Leishmania: macrophage) at 37°C. The noninternalized parasites were removed by extensive washing with sterile PBS. Then, infected cells were maintained in an incubator at 37°C and 5% CO_2_ until the moment of stimulation.

### 2.5. In Vitro Stimulus and Inhibitor Treatments

Infected macrophages (48 h after infection) were treated with 10 *μ*M Z-YVAD-FMK, 2 *μ*M Z-LEVD-FMK, or IL-1Ra (100 ng/mL) for 30 minutes before stimulation with UTP (100 *μ*M) or ATP (50, 100, or 500 *μ*M) for an additional 30 minutes at 37°C and 5% CO_2_. Then, cell monolayers were washed with PBS and maintained in DMEM supplemented (10% FBS and 100 units penicillin/streptomycin) at 37°C (and 5% CO_2_) for 24 h, when infection index and cytokine production were measured.

### 2.6. Macrophage Infection Index

Intracellular parasite loads were analyzed as previously described [19]. Briefly, cells were infected and treated with nucleotides and were fixed onto slides, stained using a panoptic stain (Laborclin®, PR, Brazil), and counted using a Primo Star light microscope (Zeiss, Germany), with a 40x objective (100x for representative pictures). Images were acquired using a Bx51 camera (Olympus, Tokyo Japan) operated using the Cell^F software. We calculated the “infection index,” representing the overall infection load, based on the count of about 100 cells in a total of five fields to obtain the number of infected macrophages and the average number of parasites per macrophage. Individual amastigotes were clearly visible in the cytoplasm of infected macrophages. The results were expressed as the infection index II = (%infected macrophages) × (amastigotes/infected macrophage)/100.

### 2.7. Murine Model of Infection and In Vivo Treatments

Female BALB/c mice, wild-type (WT), CASP-1,11^−/−^, and CASP-11^−/−^ C57BL/6 (8–12 weeks old) were infected in the dermis of the footpad by intradermal injection of 10^6^*L. amazonensis* promastigotes in PBS. Intralesional treatment with 20 *μ*L of 10 *μ*M 2-thio-UTP, 1 mM UTP (pH = 7.4), or vehicle (for 3 weeks, twice a week) started 7 days postinfection (d.p.i.). Lesion growth was calculated by evaluation of the “swelling” (thickness of the infected footpad − thickness of the uninfected footpad from the same mouse), using a traditional caliper (Mitutoyo®). Forty-eight hours after the final injection (26 d.p.i.), the animals were euthanized, and the infected footpad and popliteal lymph nodes were removed and dissociated (in M199-supplemented culture medium) for parasitic load determination.

### 2.8. Parasite Number in Mouse Tissues

The parasite load of *L. amazonensis* in infected tissues was determined using a limiting dilution assay, as previously described [19, 23]. Mice were euthanized in a CO_2_ chamber, followed by cervical dislocation. The footpads and lymph nodes were collected and weighed, and cells from the whole footpad and draining lymph nodes were dissociated using a cell strainer 40 *μ*m (BD®) in PBS. Large pieces of tissue debris were removed by centrifugation at 150 g; cells were separated by centrifugation at 2,000 g for 10 min and resuspended in supplemented M199. Samples were cultured in 96-well flat-bottom microtiter plates (BD®, USA) at 26–28°C. After a minimum of 7 days, wells were examined using phase-contrast microscopy in an inverted microscope (NIKON TMS, JP) and scored as “positive” or “negative” for the presence of parasites. Wells were scored “positive” when at least one parasite was observed per well.

### 2.9. Cytokine Levels

Measuring IL-1*β* released in cell supernatants, peritoneal macrophages from WT, and CASP-1,11^−/−^ mice were plated in 96-well plates (2.0 × 10^5^) and infected with promastigotes of *L. amazonensis* (MOI 10 : 1) as described in Section 2.4. Then, cells were treated with 100 *μ*M UTP or 3 mM ATP for 30 min. Cells were washed after 30 min and maintained at 37°C for an additional 4 h, and the supernatants were collected for further analyses. Enzyme-linked immunosorbent assays (ELISA) were performed using commercial kits, as instructed by the manufacturer (R&D Systems, Minneapolis, MN, USA). We also measured cytokine production in footpads from WT, CASP-1,11^−/−^, and CASP-11^−/−^ mice. Briefly, the infected footpads were collected and processed as described above. IL-1*β* and IL-1*α* levels were measured using ELISA with commercial kits, as instructed by the manufacturer (R&D Systems, Minneapolis, MN, USA). Protein concentrations were determined using the bicinchoninic acid method (Thermo Fisher, BCA protein assay kit, Rockford, IL, USA), and cytokine levels in tissue were corrected for by the total amount of protein.

### 2.10. Statistical Analysis

Statistical analyses were performed using the Student's *t*-test to compare two groups. For more than two groups, data were analyzed using the one-way analysis of variance (ANOVA) followed by Tukey's multiple comparison post hoc test, using the Prism 5.0 software (GraphPad Software, La Jolla, CA, USA). Differences between experimental groups were considered statistically significant when *P* < 0.05.

## 3. Results

### 3.1. P2Y_2_R Contributes to *L. amazonensis* Infection Control

**Figure 1:**
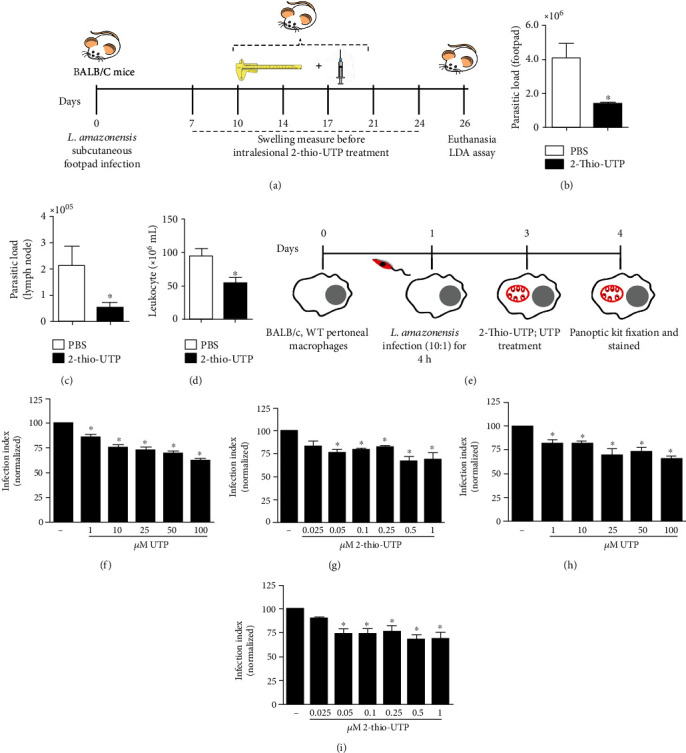
P2Y_2_R selective agonist 2-thio-UTP improves host resistance against *L. amazonensis*. (a) Schematics showing the animal model of *in vivo* experiments. BALB/c mice (*n* = 6/group) were subcutaneously injected in the footpad with 10^6^ promastigotes (*L. amazonensis* at stationary phase). From 7 days postinfection (d.p.i.), mice were treated with 10 *μ*M 2-thio-UTP in 20 *μ*L PBS and injected into the infected footpad twice a week for 3 weeks (six doses). (b–d) Animals were euthanized 26 d.p.i., and the footpads and popliteal lymph nodes were removed and used for further analysis. (b) Parasitic loads in the footpads and lymph nodes (c) were determined using a limiting dilution assay (LDA). (d) Leukocyte numbers from popliteal lymph nodes. (e) Schematics showing the design of *in vitro* experiments. BALB/c (f, g) and C57Bl/6 (WT) (h, i) macrophages infected for 48 h were treated for 30 min with UTP (1–100 *μ*M UTP) (f–h) or 2-thio-UTP (0.025–1 *μ*M) (g–i). After 30 h, cells were fixed, stained with the panoptic kit, and observed with light microscopy. The effect of treatments on infection was quantified by determining the “infection index” (%of infection × number of amastigotes/total number of cells)/100; normalized to the untreated), by direct counting under the light microscope. Data represent mean ± SEM of three independent experiments performed in triplicate, with pools of cells from four animals each experiment. ^∗^*P* < 0.05 relative to the untreated group (one-way analysis of variance followed by Tukey's test).

We recently reported that UTP-intralesional treatment elicited a Th_1_ immune response in an experimental model of cutaneous leishmaniasis [21], suggesting the involvement of P2Y_2_R. Here, we evaluated whether the intralesional treatment with a selective P2Y_2_R agonist (2-thio-UTP) would promote *L. amazonensis* control in BALB/c mice. As shown in Figure 1(a), tissues were harvested for analysis at 26 d.p.i. We found that 2-thio-UTP treatment reduced the parasitic load in the footpads (Figure 1(b)) and draining lymph nodes (Figure 1(c)), as well as the number of leukocytes in the draining lymph nodes of infected mice (Figure 1(d)) when compared to those of control mice. We also investigated the antiparasitic effect attributed to 2-thio-UTP (range 0.025–1 *μ*M) and UTP (range 1–100 *μ*M) in infected macrophages. We found that all concentrations of UTP significantly reduced the infection index in both BALB/c (Figure 1(f)) and C57BL/6 WT macrophages (Figure 1(h)). Antiparasitic effects of 2-thio-UTP treatment were observed at concentrations ranging from 0.05 to 1 *μ*M in both BALB/c (Figure 1(g)) and C57BL/6 WT macrophages (Figure 1(i)). These results suggest that P2Y_2_R activation contributes to the control of *L. amazonensis* infection.

### 3.2. Caspase-1 Is Required to P2Y_2_R-Mediated *L. amazonensis* Control in Macrophages

**Figure 2:**
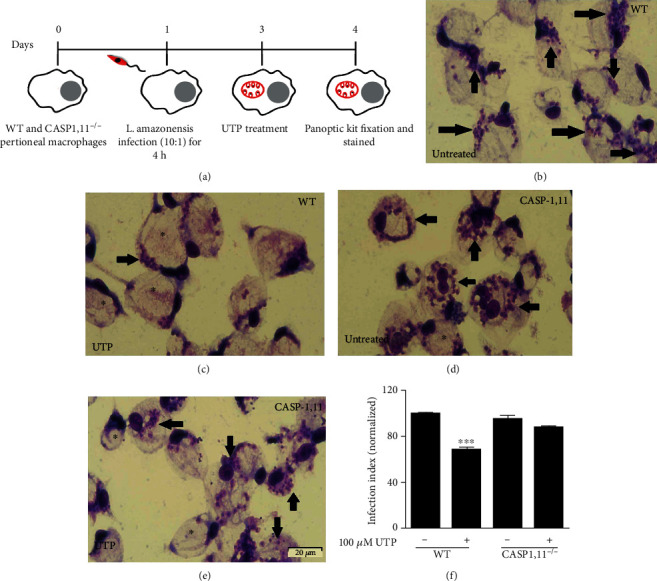
Control of *L. amazonensis* infection by UTP requires CASP-1,11. (a) Schematics showing the infection and treatments. Representative images of infected peritoneal macrophages from WT (b, c) and CASP-1,11^−/−^ (d, e) treated with UTP (100 *μ*M) (d, e) for 30 min or left untreated (b, c). The antiparasitic effect of UTP treatment was evaluated through the “infection index” (f). Data represent mean ± SEM of three independent experiments performed in triplicate, with pools of 3–4 animals in each experiment. ^∗∗∗^*P* < 0.0001 relative to the untreated group (one-way analysis of variance followed by Tukey's test).

**Figure 3:**
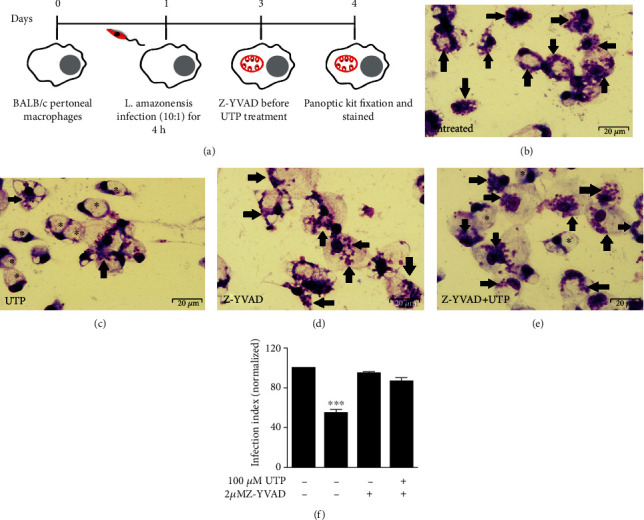
The antiparasitic effect by P2Y_2_R requires CASP-1 activity. (a) Schematics showing the infection and treatments. Infected peritoneal macrophages from BALB/c were treated with UTP (100 *μ*M) (c, e) for 30 min or left untreated (b) following Z-YVAD-FMK (2 *μ*M) (d, e). The antiparasitic effect of UTP treatment was evaluated through the “infection index” (f). Data represent mean ± SEM of three independent experiments performed in triplicate, with pools of 3–4 animals in each experiment. ^∗∗∗^*P* < 0.0001 relative to the untreated group (one-way analysis of variance followed by Tukey's test).

Previously, we showed that antiparasite effects attributed by UTP and ATP involve P2X7R and PANX-1 channels in infected macrophages [19]. We also reported that antiparasitic immune responses triggered by the ATP/P2X7R/PANX-1 axis require NLRP3 inflammasome activation in macrophages infected with *L. amazonensis* [14]. Here, we determined whether CASP-1 activation (an essential component of the NLRP3 inflammasome) participates in infection control mediated by eUTP. We found that the antileishmanial effects of UTP were absent in infected macrophages from mice genetically deficient for CASP-1,11 enzymes (CASP-1,11^−/−^ mice) (Figures 2(b)–2(f)). When we blocked CASP-1 with Z-YVAD-FMK (CASP-1 inhibitor), the antiparasitic effects attributed to UTP treatment were abrogated (Figures 3(b)–3(f)). By contrast, the antiparasitic effects of UTP were significant in macrophages from CASP-11^−/−^ mice and in WT macrophages treated with Z-LEVD-FMK (CASP-11 inhibitor) (Supplementary Figure 1). These findings suggest that the activity of CASP-1 but not CASP-11 is relevant to *L. amazonensis* control mediated by the P2Y_2_ receptor.

### 3.3. P2Y_2_R Stimulation Promotes IL-1*β* Secretion from *L. amazonensis*-Infected Macrophages

**Figure 4:**
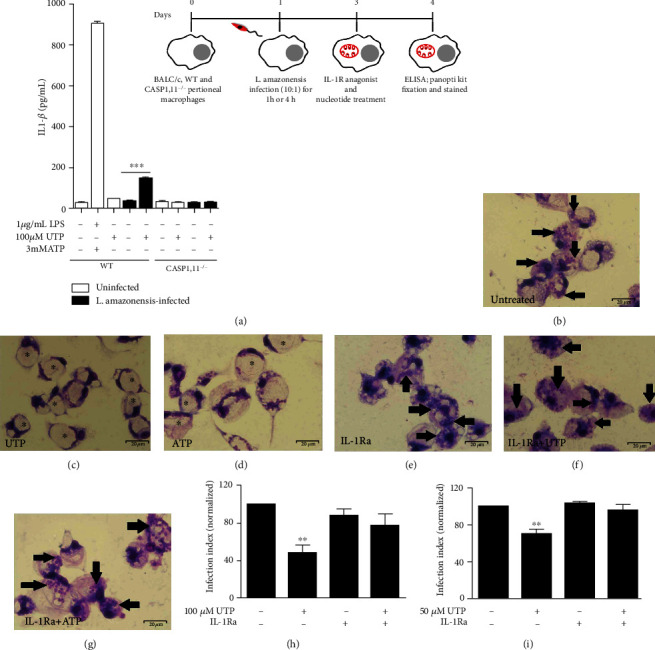
Control of *L. amazonensis* infection by UTP and ATP involves IL-1R signaling. (a) Peritoneal macrophages from WT and CASP 1,11^−/−^ mice were infected with *L. amazonensis* promastigotes for 1 h and treated with UTP for 30 min. Cell culture supernatants were harvested 4 h later. Uninfected cells were primed with LPS (1 *μ*g/mL) for 2 h followed by ATP (3 mM), as the second signal (a positive control). IL-1*β* was measured 6 h later in the positive control group. (b–d) BALB/c-infected macrophages untreated (e–g) or treated with IL-1Ra (100 ng/mL) for 30 min prior (c, f) UTP and (d, g) ATP pulse for 30 min. Twenty-four hours after nucleotide treatment, cells were fixed and stained using the panoptic kit to measure parasitic load using the “infection index” (h, i). Data are mean ± SEM of three independent experiments performed in triplicate, with pools of 3–4 animals in each experiment. ^∗∗∗^*P* < 0.0001 and ^∗∗^*P* < 0.005 relative to the untreated group (one-way analysis of variance followed by Tukey's test).

IL-1*β* is an important proinflammatory cytokine produced in response to several pathogens, and its secretion is induced by inflammasome activation in a P2X7-dependent manner during *L. amazonensis* infection [24]. Therefore, we determined whether activation of the inflammasome via UTP/P2Y_2_R would result in the secretion of IL-1*β* by *L. amazonensis*-infected cells. We found that UTP treatment induced IL-1*β* secretion in WT-infected macrophages but not in macrophages from CASP-1/11^−/−^ mice (Figure 4(a)), suggesting that activation of the canonical NLRP3 inflammasome by eUTP triggers IL-1*β* secretion in *L. amazonensis*-infected macrophages in a caspase-1-dependent fashion. Treatment with UTP or ATP did not reduce the parasitic load in infected macrophages pretreated with IL-1R antagonist (Figures 4(b)–4(i)), suggesting that IL-1R signaling is essential to *L*. *amazonensis* control mediated by P2X and P2Y receptors.

### 3.4. Treatment with UTP Promotes the Control of In Vivo *L. amazonensis* Infection through Caspase-1 Activation and IL-1*β* Production

**Figure 5:**
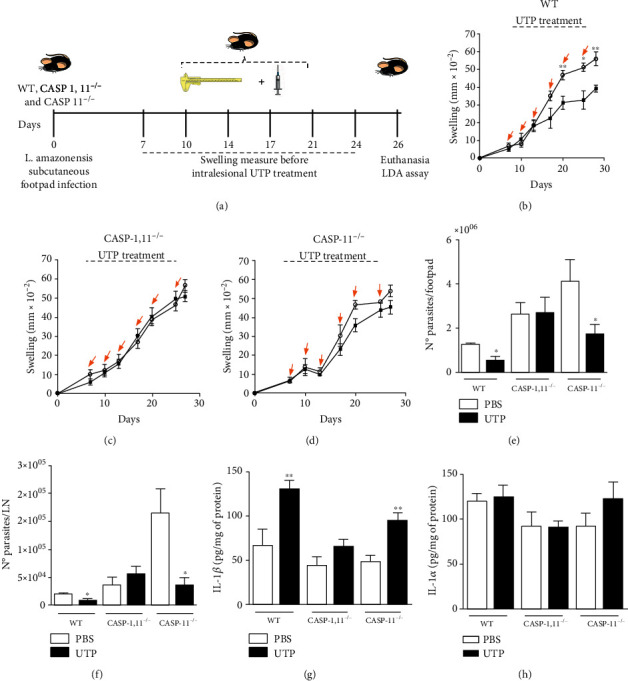
CASP-1 activity is necessary for the protective effects of UTP during *in vivo* infection with *L. amazonensis*. (a) Schematics showing the experimental approach of *in vivo* experiments. WT, CASP-1,11^−/−^, and CASP-1,11^−/−^ mice (*n* = 5 and 6/group) were infected with 10^6^*L. amazonensis*; following 7 d.p.i., we started the intralesional treatment with 1 mM UTP into the infected footpad twice a week for 3 weeks (six doses). (b–d) Swelling (thickness) was measured using a traditional Mitutoyo® caliper, and the lesion size was determined by the thickness of the infected footpad − thickness of the uninfected footpad from the same mouse. (e–h) Animals were euthanized 26 d.p.i. when the footpad and popliteal lymph nodes were excised and used to quantify parasite load and cytokine production. (e) Parasite loads in the footpads (f) and in popliteal lymph nodes from WT and CASP 1,11^−/−^ mice by limiting dilution assay (LDA). (g) IL-1*β* and (h) IL-1*α* production into the footpad was measured by ELISA. Data represent mean ± SEM values, *n* = 5–6 mice per group. ^∗∗^*P* < 0.01 and ^∗^*P* < 0.05 in comparison to the untreated group (one-way analysis of variance followed by Tukey's test).

To confirm the importance of the CASP-1 activation by UTP/P2Y_2_ activation, we evaluated the requirement of CASP-1 in the protection elicited by UTP treatment during *L. amazonensis* infection *in vivo*. We injected UTP at intervals of 3–4 days, from 7 days postinfection in the footpads of WT, CASP-1,11^−/−^, and CASP-11^−/−^ mice infected with 10^6^ promastigotes of *L. amazonensis*. Mice were euthanized 26 days postinfection, and lesion development (swelling) was measured during the development of leishmaniasis (Figure 5(a)). As depicted in Figure 5(b), WT mice treated with UTP showed significantly smaller lesion sizes and lower parasite loads (the final one both in the footpads and lymph nodes) than in control mice (Figures 5(b), 5(e), and 5(f)). However, these protective effects were absent in CASP-1,11^−/−^ mice, where UTP treatment did not reduce either lesion size or parasite load (Figures 5(c), 5(e), and 5(f)). UTP treatment in CASP-11^−/−^-infected mice did not significantly decrease lesion size but rather induced a significant reduction in parasite load (Figures 5(d), 5(e), and 5(f)). Of note, both knockouts showed increased parasite loads when compared to infected WT mice (Figures 5(e) and 5(f)), as previously reported [15].

We also evaluated whether CASP-1 and CASP-11 were relevant to the production of IL-1*β* and IL-1*α* in mice treated with UTP. The footpads from infected WT and CASP-11^−/−^ mice treated with UTP showed higher levels of IL-1*β*, as compared to the control footpads (Figure 5(g)). However, no increase in IL-1*β* was found in the footpads of UTP-treated CASP-1,11^−/−^ mice (Figure 5(g)), suggesting that CASP-1 is involved in UTP-induced IL-1*β* production. Levels of IL-1*α* did not differ in the footpads from WT, CASP-1,11^−/−^, and CASP-11^−/−^ mice after UTP treatment (Figure 5(h)).

## 4. Discussion

Cutaneous leishmaniasis affects millions of people worldwide. Nevertheless, the host defense mechanisms that are modulated to control parasite replication and treat the disease are not thoroughly characterized, and several aspects of the disease remain poorly understood [25]. We previously reported the involvement of purinergic receptors, including P2Y_2_R and P2X7R, in the control of *L. amazonensis* infection *in vitro* and *in vivo* [4, 19]. These receptors induce the activation of several microbicidal mechanisms in host cells during infection (i.e., NO, ROS, LTB_4_ production) [20, 21, 26, 27]. Here, we identified a protective mechanism triggered by P2Y_2_R, using incubation with either a specific agonist for P2Y_2_R, 2-thio UTP, or low concentrations of UTP (100 *μ*M) or ATP (50 *μ*M) during *in vitro* infection with *L. amazonensis*. Parasite elimination upon P2Y_2_R activation *in vitro* and *in vivo* depends on caspase-1 activation and IL-1R signaling.

The role of P2X7R in the IL-1*β* maturation via NLRP3 inflammasome assembly in both infectious and inflammatory disorders is currently accepted [16, 28, 29]. Jin et al. [10] also proposed a P2Y_2_R-mediated inflammasome activation pathway [10]. Interestingly, inflammasome assembly and signaling are closely associated with the physiopathology of leishmaniasis [30–32]. We and others have shown that the NLRP3 inflammasome is protective and contributes to restricting *L. amazonensis* parasite replication in macrophages as well as *in vivo* [30, 33, 34]. The canonical NLRP3 inflammasome promotes IL-1*β* and IL-18 activation through the engagement of NLRP3, ASC, and caspase-1 activation. In addition to these components, the noncanonical NLRP3 inflammasome requires a caspase-11 expression for proper caspase-1 activation [35].

Despite the apparent importance of the inflammasome for disease outcomes, the mechanisms by which NLRP3 inflammasome is activated during *Leishmania* infection remain poorly understood. ROS production via dectin-1 and P2X7R activation appears to be involved in NLRP3 inflammasome activation [14, 34]. The mechanisms of NLRP3 inflammasome activation required for IL-1*β* generation during infection by *Leishmania* have been recently elucidated. IL-1*β* from a noncanonical NLRP3 source was implicated in the elimination of pathogens [36, 37] including *L. amazonensis* [14]. In support of this finding, caspase-11 was shown to be activated in response to the cytosolic delivery of *Leishmania* LPG in macrophages [30]. Previous *in vitro* studies from our group reported that ATP/P2X7 axis was critical for *L. amazonensis* control in a mechanism dependent on IL-1R signaling by noncanonical NLRP3 inflammasome activation [14, 24]. In these settings, *L. amazonensis* elimination by P2X7R activation was followed by LTB_4_ release and subsequent activation of the NLRP3 complex and IL-1*β* release, requiring the activation of both CASP-1 and CASP-11 [4, 14]. NLRP3 activation by LTB_4_ depended on ROS induction [38]. Likewise, P2X7R activation triggered ROS production and the host immune response in several intracellular pathogen diseases, including *Toxoplasma gondii*, *Chlamydia* spp., and *Plasmodium chabaudi* infections [39–41].

P2X7R was also necessary for antiparasitic responses attributed to UTP/P2YR in *L. amazonensis* infection [19]. The elimination of *L. amazonensis in vitro* triggered by P2Y_2_R involves P2X7-dependent LTB_4_ secretion, in a mechanism requiring pannexin-1. P2X7R expression was also required for *in vivo* control of *L. amazonensis* by UTP, supporting the notion of a collaborative effect among P2 receptors to improve the antiparasitic immune response in leishmaniasis [19]. In the present study, we showed, through *in vitro* and *in vivo* studies, that CASP-1 and IL-1R signaling, but not CASP-11, were necessary to boost host immune responses induced by P2Y_2_R, contributing to protection against *L. amazonensis*.

Antileishmanial effects triggered by P2Y_2_R involve two steps. The first one is mediated by ATP release with an autocrine/paracrine effect on P2X7R [19]. The second reveals a pathway of IL-1*β* induction triggered by P2Y_2_R (activated by low ATP concentrations). Corroborating our results, P2YR signaling triggered inflammasome activation in several inflammatory models [9, 13], and caspase-1-mediated secretion of IL-1*β* after activation of NLRP3 [9] and NLCR4 inflammasomes [42] after P2Y_2_R activation have been reported.

In the present study, we showed that P2Y_2_R induced lower levels of extracellular IL-1*β* when compared to P2X7R engagement by exogenous ATP, even though the technique used to measure extracellular nucleotides did not directly reflect their concentration around cells. These findings suggest that the P2X7R-dependent pathway triggered by P2Y_2_R could be activated to a lesser extent than that of P2X7R activation triggered by exogenous ATP. P2X7 receptor activation and its effect on immune cells vary according to ATP levels. P2X7 receptor stimulation with low ATP (100 *μ*M) leads to the formation of a cation-selective channel. Its activation by high levels of ATP triggers the formation of a nonselective pore [43, 44]. The latter scenario is usually related to pronounced ROS production, inflammasome activation, and IL-1*β* release in various systems [45]. High ATP levels can activate the P2X7R that acts as the second signal for noncanonical NLRP3 inflammasome activation during *L. amazonensis* infection. Chaves et al. [14] demonstrated activation of the noncanonical NLRP3 and IL-1*β* release after P2X7R activation *in vitro* after incubation of infected macrophages with exogenous ATP (500 *μ*M) [14]. In this context, P2X7R activation by high exogenous ATP concentrations during infection with *L. amazonensis* might lead to higher levels of extracellular LTB_4_, which in turn would favor ROS production and triggering of the noncanonical NLRP3 inflammasome assembly. Our data suggest that P2Y_2_ receptor activation is not able to induce P2X7 receptor activation at a level that triggers activation of the noncanonical NLRP3 inflammasome assembly. Nevertheless, the exact inflammasome-related pathways triggered under these conditions are unknown and require further studies.

Our *in vivo* results showed that, while lack of CASP-1/11 abrogated IL-1*β* induction and reduction in parasite load after activation of P2Y_2_R, mice deficient in CASP-11 preserved an antileishmanial response after treatment with UTP. These findings suggest that, during *in vivo* infection by *L. amazonensis*, caspase-1 is the main source of IL-1*β* and is responsible for controlling the parasitic infection. This pathway is further enhanced by P2Y_2_R signaling. Even though P2X7R activation appears to enhance IL-1*β* production through the noncanonical NLRP3 *in vitro* [14], its participation in the control of *in vivo* infection by *L. amazonensis* has not been addressed. It needs to be studied in depth before reaching further conclusions. Taken together, these data suggest that not only P2X7R but also P2Y_2_R, a G-protein-coupled receptor, is involved in the activation of IL-1*β* production/release in immune cells during infection with *L. amazonensis*. IL-1R activation, in turn, controls *L*. *amazonensis* infection. These results suggest that DAMPs boost the immune response against *L. amazonensis* infection via P2 receptors.

## 5. Conclusion

P2Y_2_R activation by UTP/ATP induced CASP-1 activation and IL-1*β* secretion in the context of *L. amazonensis* infection. IL-1*β*/IL-1R signaling was crucial to P2Y_2_R-mediated protective immune responses in an experimental model of cutaneous leishmaniasis. These findings suggest that P2Y_2_R may be a possible therapeutic target to treat *L. amazonensis* infection by potentiating IL-1*β*/IL-1R signaling and controlling parasite replication.

## Data Availability

The file data used to support the findings of this study are available from the corresponding author upon request.

## Supplementary Materials

Supplementary Materials Supplementary Figure 1: infected peritoneal macrophages from BALB/c (A) were pretreated with Z-YVAD-FMK (2 *μ*M) for 30 minutes and then with 100 *μ*M UTP for another 30 minutes. Peritoneal macrophages from WT or CASP-11^−/−^ were infected and after 48 h treated or not with UTP (100 *μ*M) for 30 minutes (B). Twenty-four hours later, cells were fixed and stained with panoptic stain, and glass coverslips on slides were evaluated using the “infection index” by a direct count under light microscopy. Data represent mean ± SEM of three independent experiments performed in triplicate, with pools of 3–4 animals in each experiment. ^∗∗∗^*P* < 0.0001 relative to the untreated group (one-way analysis of variance followed by Tukey's test).
