# Supplementary figures and images for: P2Y2 Receptor Induces L. amazonensis Infection Control in a Mechanism Dependent on Caspase-1 Activation and IL-1β Secretion
Source: Mediators Inflamm. 2020 Oct 1;2020:2545682. doi: 10.1155/2020/2545682 (PMC7547346; doi:10.1155/2020/2545682)

**A**

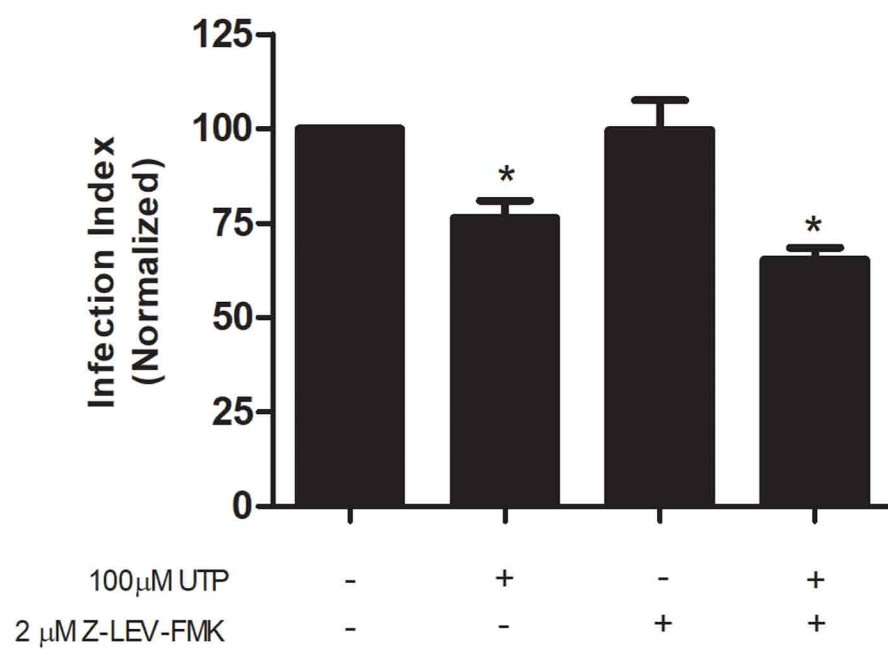

**B**

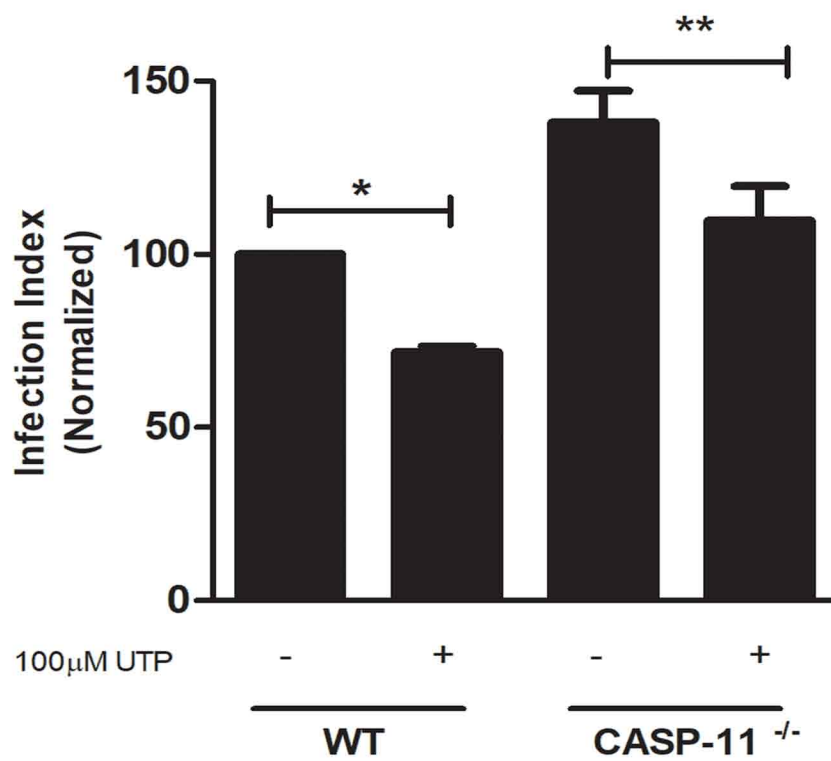

Supplement: Supplementary Materials — Supplementary Figure 1: infected peritoneal macrophages from BALB/c (A) were pretreated with Z-YVAD-FMK (2 μM) for 30 minutes and then with 100 μM UTP for another 30 minutes. Peritoneal macrophages from WT or CASP-11−/− were infected and after 48 h treated or not with UTP (100 μM) for 30 minutes (B). Twenty-four hours later, cells were fixed and stained with panoptic stain, and glass coverslips on slides were evaluated using the “infection index” by a direct count under light microscopy. Data represent mean ± SEM of three independent experiments performed in triplicate, with pools of 3–4 animals in each experiment. ∗∗∗P < 0.0001 relative to the untreated group (one-way analysis of variance followed by Tukey's test). [file 2545682.f1.pdf]
